# Empowering the Future: Transitioning to Adulthood With Congenital Heart Disease

**DOI:** 10.1016/j.cjcpc.2023.09.007

**Published:** 2023-09-19

**Authors:** Philip Moons

**Affiliations:** a Department of Public Health and Primary Care, KU Leuven, Leuven, Belgium; b Institute of Health and Care Sciences, University of Gothenburg, Gothenburg, Sweden; c Department of Paediatrics and Child Health, University of Cape Town, Cape Town, South Africa

“I must say, about the consequences… oh my god how they spoke about drinking and sex at my last visit! - If you drink alcohol you’re going to die or end up in an emergency room. You are not allowed to get drunk… and if you have unprotected sex you’re going to die… oh my god…it was like they had to tell me everything, it was horrible! But hello, I’m not stupid…it was too much, I was devastated… You have to individualize such information…!”

This excerpt is a quotation from an 18-year-old girl diagnosed with congenital heart disease (CHD) when queried about her encounters with the information dispensed before her transfer from paediatric cardiology to adult care.^1^ It is well known that adolescents with CHD possess limited knowledge concerning their cardiac condition and the requisite self-care practices.^2^^,^^3^ One pivotal factor contributing to this knowledge gap is the tendency to primarily educate and inform parents during childhood, thus inadvertently sidelining the vital role of the afflicted adolescents themselves. However, as these children progress into adolescence, they naturally yearn for and require active involvement in the communication process. As they embark on the journey toward assuming greater responsibility for their own health, it becomes imperative for them to cultivate a profound comprehension of how to maintain good health and guard against potential complications.

To enhance the knowledge base of adolescents and young adults with CHD, patient education plays a pivotal role. Research has demonstrated the effectiveness of patient education in elevating the knowledge levels of people with CHD.^4^, ^5^, ^6^ To bolster this impact, patient education is embedded in structured transition programmes. These programmes are defined as “a set of co-ordinated transitional care interventions that are provided in a structured albeit individualized way, in order to support the process of the transition to adulthood and achieve the outcomes of transition.”^7^ There is an accumulating body of evidence indicating that transition programmes augment the level of knowledge of afflicted adolescents.^8^, ^9^, ^10^, ^11^ However, it is crucial to acknowledge that improving patient knowledge alone is insufficient as an outcome of transition programmes. International consensus panels have suggested a comprehensive set of key outcomes to characterize a successful transition. These encompass aspects such as quality of life, self-management, disease knowledge, continuity of care, appropriate health care consumption, disease control, access to care/receiving coordinated care, interaction with adult providers, and interaction with peers.^12^^,^^13^ Transitional care interventions should be designed and implemented with these multifaceted goals as the foundation. Nonetheless, it is essential to recognize that these objectives often constitute distant endpoints and may not be promptly attainable. Hence, it is prudent to consider an overarching and more immediate outcome: empowerment. Empowerment has demonstrated associations with numerous other outcomes, including health status, quality of life, and self-care.^14^ From this perspective, empowerment emerges as a promising and practical outcome measure for transition programmes, given its potential to indirectly influence the achievement of more distal objectives.^7^ A recent trial in the “**S**wedish **T**ransition **E**ffects **P**roject **S**upporting **T**eenagers with chr**ON**ic m**E**dical condition**S**” (STEPSTONES) has demonstrated the effectiveness of a transition programme to empower young people with CHD.^11^

Various methodologies exist for crafting complex interventions like transition programmes. One highly effective approach is intervention mapping. Intervention mapping is a systematic method that amalgamates theoretical constructs, insights gleaned from existing literature, and input solicited from engaged stakeholders.^15^ This method allows researchers to create a blueprint of the intervention by illuminating how each component is expected to affect the outcome assessed.^16^ This approach not only ensures the theoretical robustness of the intervention but also bolsters its underpinning framework. To the best of my knowledge, the STEPSTONES transition programme stands as a unique exemplar developed through the application of intervention mapping.^17^ An alternative way to develop a transition programme hinges on the principles of co-design, also known as participatory design. This method revolves around actively engaging all relevant stakeholders in the design process, with the overarching aim of aligning the end product with its specific needs and ensuring its usability. The development of transitional care interventions lends itself particularly well to co-design, as it circumvents potential disparities between patients’ desires and requirements and the services provided by health care professionals, as elucidated by the quote cited at the beginning of this editorial. A key difference between intervention mapping and co-design is that the former does not necessarily involve patients in the developmental phase, whereas this is a critical element in co-design.

In the current issue of *CJC Pediatric and Congenital Heart Disease*, an article was published elucidating the co-design of a comprehensive transition programme for young people with CHD, encompassing essential components.^18^ The impetus driving this endeavour stemmed from the observation that existing CHD transition programmes have, to date, not directly included patients, caregivers, and health care providers as active collaborators in their development. The development described here harnessed a patient engagement framework and drew upon insights gleaned from literature reviews concerning patient engagement within the realms of health services research and quality improvement.^18^ The co-design process has unveiled pivotal facets pertaining to “The What,” “The Who,” “The When,” and “The How” of providing information, education, and support during the transition process of adolescents with CHD. It has been collectively affirmed by patients, caregivers, and health care providers that the provision of support is an imperative cornerstone, serving as a catalyst for the empowerment of patients in taking ownership of their health care and life. This support includes a spectrum of self-management skills such as monitoring symptoms, career planning, and lifestyle considerations, as well as knowledge about their specific heart defect. The responsibility for providing information and education falls on the health care team, with nurses and social workers playing crucial roles. Peer support was also considered of significant value, providing a platform for patients and caregivers to connect with others who understand the unique challenges of living with CHD.^18^

This co-designed transition programme unequivocally reinforces the imperative shift from a paradigm of mere information dissemination to one of empowerment. Health care professionals involved in the care of young people with CHD have the pivotal duty to enable and equip young persons with the tools, knowledge, resources, or confidence needed to shape and improve their future in a positive and self-determined way. It implies fostering a sense of self-efficacy, independence, and the ability to make choices that lead to better outcomes.

Should clinicians effectively facilitate the process of these young patients assuming control over their health care decisions and overall well-being as they navigate the multifaceted challenges of managing their condition in the realm of adulthood, they unequivocally contribute to “empowering the future.”
